# Comparison of innate immune agonists for induction of tracheal antimicrobial peptide gene expression in tracheal epithelial cells of cattle

**DOI:** 10.1186/s13567-014-0105-8

**Published:** 2014-10-12

**Authors:** Lesley Berghuis, Khaled Taha Abdelaziz, Jodi Bierworth, Leanna Wyer, Gabriella Jacob, Niel A Karrow, Shayan Sharif, Mary Ellen Clark, Jeff L Caswell

**Affiliations:** Department of Pathobiology, University of Guelph, Guelph, ON N1G 2 W1 Canada; Pathology Department, Faculty of Veterinary Medicine, Beni-Suef University, Beni-Suef, Egypt; Department of Animal and Poultry Science, University of Guelph, Guelph, ON N1G 2 W1 Canada

## Abstract

Bovine respiratory disease is a complex of bacterial and viral infections of economic and welfare importance to the beef industry. Although tracheal antimicrobial peptide (TAP) has microbicidal activity against bacterial pathogens causing bovine respiratory disease, risk factors for bovine respiratory disease including BVDV and stress (glucocorticoids) have been shown to inhibit the induced expression of this gene. Lipopolysaccharide is known to stimulate TAP gene expression, but the maximum effect is only observed after 16 h of stimulation. The present study investigated other agonists of TAP gene expression in primary cultures of bovine tracheal epithelial cells. PCR analysis of unstimulated tracheal epithelial cells, tracheal tissue and lung tissue each showed mRNA expression for Toll-like receptors (TLRs) 1–10. Quantitative RT-PCR analysis showed that Pam3CSK4 (an agonist of TLR1/2) and interleukin (IL)-17A significantly induced TAP gene expression in tracheal epithelial cells after only 4–8 h of stimulation. Flagellin (a TLR5 agonist), lipopolysaccharide and interferon-α also had stimulatory effects, but little or no response was found with class B CpG ODN 2007 (TLR9 agonist) or lipoteichoic acid (TLR2 agonist). The use of combined agonists had little or no enhancing effect above that of single agonists. Thus, Pam3CSK4, IL-17A and lipopolysaccharide rapidly and significantly induce TAP gene expression, suggesting that these stimulatory pathways may be of value for enhancing innate immunity in feedlot cattle at times of susceptibility to disease.

## Introduction

Tracheal antimicrobial peptide is an inducible β-defensin that is highly expressed in the bovine respiratory tract and has demonstrated antimicrobial effects on pathogens that cause bovine respiratory disease, including *Mannheimia haemolytica*, *Histophilus somni* and *Pasteurella multocida* [1-3]. TAP expression is induced by several pro-inflammatory cytokines as well as bacterial components such as lipopolysaccharide (LPS) [4-7]. However, infection with certain viruses and bacteria, or exposure to glucocorticoids, inhibits this response to LPS, implying that this may be one mechanism by which stress and viral infection increase the risk of bacterial pneumonia in cattle [8-11]. Importantly, the susceptibility of bovine bacterial pathogens to TAP suggests that restoring this suppressed response may be a viable method to prevent disease in stressed or virus-infected cattle [11].

The induction of TAP gene expression by LPS is delayed and does not reach zenith until 16 h of stimulation, perhaps suggesting indirect signal transduction. Thus, the purpose of this study was to determine which TLR receptors are constitutively expressed by bovine tracheal epithelial cells, and to identify agonists that induce high levels of TAP gene expression more rapidly than LPS. Toll-like receptor (TLR) ligands were a focus of the study, including lipoteichoic acid (TLR2/2), Pam3CSK4 (TLR2/1), FSL-1 (TLR2/6), LPS (TLR4), flagellin (TLR5), and CpG ODN 2007 (TLR9). We also examined IL-17A because of its emerging recognition as a critical stimulus for mucosal defence against bacterial infection, and interferon (IFN)-α because of the commercial availability of an immunostimulant (inactivated Parapoxvirus ovis, Zylexis) known to induce its expression [12].

## Materials and methods

### Toll-like receptor and IL-17A receptor subunit A gene expression

Tracheal and lung tissue from three clinically and pathologically normal market-weight calves were obtained at the time of slaughter. From each calf, 30 mg samples of tracheal mucosal tissue and lung tissue were washed in PBS and preserved in RNAlater® at 4 °C for 24 h prior to storage at −80 °C. Adjacent samples of the same tissues were fixed in formalin for 48 h and histologic sections were prepared routinely. Histologically, the tracheal and lung samples from all calves were within normal limits and had only low numbers of lymphocytes, plasma cells, and few neutrophils.

Fresh tracheas were obtained as above and primary cultures of bovine tracheal epithelial cells (bTEC) were prepared as previously described [10]. Briefly, cells were cultured for 4–6 days until 80-90% confluency in a 1:1 mixture of Dulbecco’s modified Eagle’s medium and Ham’s F-12 medium containing 10% fetal bovine serum, 0.1 mg/mL penicillin-streptomycin, 5 μg/mL amphotericin B, 0.5 mg/mL cell culture grade gentamycin, 25 μg/mL bovine pituitary extract, 25 ng/mL epidermal growth factor and 1% insulin-transferrin-selenium. To confirm the epithelial nature of the cells, immunohistochemistry was performed on cells fixed in 70% ethanol and suspended in HistoGel™ (Thermo Fisher Scientific Inc.) for pancytokeratin (AE1/AE3, M3515, Dako, ON, Canada) and vimentin (M0725, Dako); negative control sections were processed in the same way with omission of the primary antibody. RNA was extracted from tissues preserved in RNA later® and from cultured bTEC using RNAeasy® Mini Kit capture columns (QIAGEN Inc, Mississauga, ON, Canada) and QIAshredder™ (QIAGEN Inc) according to manufacturer’s instructions. Genomic DNA was removed by treatment with DNase I (QIAGEN Inc). The concentration and quality of extracted RNA was measured using a Nanodrop 2000 spectrophotometer (Thermo Scientific; Wilmington, DE, USA). Complementary single-stranded DNA (cDNA) was reverse transcribed from 100 ng total RNA using Super Script® III reverse transcriptase (Thermo Scientific) according to the manufacturer’s protocol using oligo dTs as the primer. Once cDNA synthesis was completed, samples were incubated at 37 °C for 20 min with ribonuclease H (Invitrogen) to remove any remaining RNA.

PCR amplification of cDNA was completed using primers for TLRs 1–10 [13,14]. For IL-17RA, sequences were obtained from NCBI (XM_002683626.2) and primers were designed using NCBI primer designer software: forward primer 5’-GTGCCCGACTGCAAGGACCC-3’, reverse 5’-CTGGGTGGCTTCCTGTGCGG-3’, 250 bp product size. The PCR profile was as follows: 95 °C for 5 min, 95 °C for 30 s, 58 °C for 30 s for TLR primers and 65 °C for 30 s for IL-17RA primers, 72 °C for 30 s, repeat from step 2 for 35 cycles, 72 °C for 5 min. The PCR products were analyzed by gel electrophoresis using 0.01% SYBR® Safe gel dye on a 5% agarose gel (Sigma, St. Louis, MO, USA) in 0.5× Tris/Borate/EDTA buffer (Sigma). Product sizes were compared to a 100 bp ladder, and products of each reaction were confirmed once by sequencing. Pooled cDNA from mesenteric lymph node and ileum obtained from a healthy Holstein calf were used as positive controls for the TLR gene expression study, because these tissues were previously shown to express all 10 bovine TLRs [13]. Negative controls consisted of PCR reactions that included receptor-specific primer pairs but contained no template cDNA.

### Effect of agonists on TAP gene expression

Confluent bTEC, cultured as described above, were incubated with serum-free medium (DMEM/F12, 0.1 mg/mL penicillin-streptomycin and 1.5 μg/mL amphotericin B) at 37 °C in 5% CO_2_ for 6–12 h, then incubated in triplicate with various TLR agonists or cytokines. The effect of different doses of each agonist was examined first, with subsequent testing of the time course needed to induce gene expression at the optimal doses. The TLR agonists included lipoteichoic acid from S*taphylococcus aureus* (an agonist of TLR2/2 homodimer; Sigma-Aldrich, St. Louis, MO, USA, item number L2630), Pam3CSK4 (agonist of TLR2/1 heterodimer; Invivogen, San Diego, CA, item t1rl-pms), FSL-1 (agonist of TLR2/6 heterodimer; Invivogen, item t1r1-fsl), flagellin from *Salmonella enteritica* serovar Typhimurium (TLR5 agonist; Invivogen, San Diego, CA, item t1r1-pstfla), and class B CpG ODN 2007 (bovine/porcine-specific TLR9 agonist; Invivogen, item t1rl-2007) along with the negative control of non-CpG ODN 2007 (consisting of GpC motifs; Invivogen, item t1r1-2007c). The cytokines included bovine recombinant IL-17A (Kingfisher Biotech, St. Paul, MN, USA, item number RP0056B-005) and bovine recombinant IFN-α (Kingfisher Biotech, item RP0008B-025). Lipopolysaccharide derived from *Pseudomonas aeruginosa* (TLR4 agonist; Sigma Aldrich, MO, USA, item L9143), was included as a standard and positive control in each trial, at a concentration of 0.1 μg/mL that had been previously optimized [10].

To quantify relative gene expression, real-time reverse transcription quantitative PCR was conducted using LightCycler® 480 technology (Roche Diagnostics, Mannheim, Germany). Constant amounts of cDNA synthesized from 100 ng of total RNA were used in each reaction to measure gene expression of TAP relative to the reference gene glyceraldehyde-3-phosphate dehydrogenase (GAPDH). The GAPDH Ct values remained within 1 threshold cycle across various treatments, confirming constant gene expression under the conditions tested. The primer sequences and product sizes for TAP and GAPH were as follows: TAP forward 5’-TCTTCCTGGTCTGTCTGCT-3’, reverse 5’-GCTGTGTCTTGGCCTTCTTT-3’, 183 bp product size; GAPDH forward 5’-GGCGTGAACCACG AGAAGTATAA-3’, reverse 5’-CCCTCCACGATGCCAAAGT-3’, 120 bp product size [10]. Amplification efficiencies of 1.91 for TAP and 1.88 for GAP were determined from the slope of a standard curve constructed from a series of 1:5 serial dilutions of cDNA synthesized from LPS-stimulated bTEC. A middle dilution was used in each qPCR run as a calibrator to control for plate-to-plate variation. Each qPCR reaction consisted of 10 μL of 2× LightCycler®480 SYBR Green 1 master mix (Roche Diagnostics, Mannheim, Germany), 0.5 μL each of forward and reverse primers (10 μM), 7 μL of PCR grade water and 2 μL of 1:10 diluted template cDNA. The reaction was started by a pre-incubation step at 95 °C for 7 min followed by 45 amplification cycles consisting of 95 °C for 15 s, 63 °C for 15 s, and 72 °C for 20 s. Once completed, a melting curve was produced at 95 °C for 5 s then 45 °C for 1 min. The reaction was concluded by a cooling step at 40 °C for 1 min. The samples were run in technical duplicates for both the gene of interest (TAP) and the reference gene (GAPDH) then averaged for advanced relative quantification analysis using LightCycler®480 software (Roche Diagnostics, Mannheim, Germany). Two types of negative controls were used in each trial: a non-stimulation control that differed from the other wells only in the absence of an agonist, and an additional negative control containing master mix but no template cDNA.

### Statistical analysis

A general linear mixed model was used to fit the normalized ratios using Proc MIXED (SAS 9.2). Fixed effects were a combination of agonist and dose by time and the combination by time interaction were also included in the model. Calf identification was a random blocking variable (a random effect), and the random interaction of calf identification by dose-agonist combination by time was used to handle sub-sampling (biologic triplicates). To assess the ANOVA assumptions, residual analyses were conducted. The residuals were formally tested for normality using the four tests offered by SAS (Shapiro-Wilk, Cramér-von Mises, Kolmogorov-Smirnov and Anderson-Darling tests). In addition, the residuals were plotted against the predicted values and explanatory variables included in the model. Log transformation and exclusion of 2 outliers produced a fundamentally normal distribution of data. *P* < 0.05 was considered significant. The data are shown graphically as mean ± standard error of the mean (SEM).

## Results

### Toll-like receptor and IL-17A receptor subunit A gene expression in bovine respiratory cells and tissue

**Figure 1:**
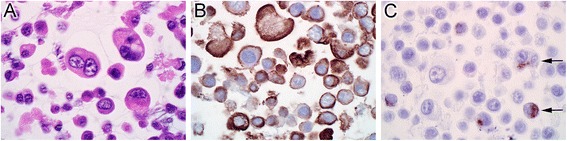
**Histologic appearance of trypsinized primary cell cultures of bovine tracheal epithelial cells. A)** Hematoxylin and eosin stain. **B)** Immunohistochemistry for pancytokeratin. **C)** Immunohistochemistry for vimentin, with immunolabeled cells shown by arrows.

**Figure 2:**
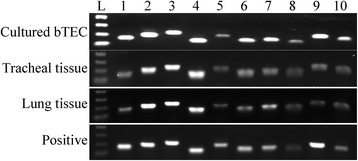
**Expression of Toll-like receptor (TLR) in tracheal epithelial cells, tracheal mucosa, and lung.** Basal expression of mRNA expression for TLRs 1–10 was evaluated in cultured bovine tracheal epithelial cells, tracheal mucosal tissue, lung tissue, and positive control tissue (pooled mesenteric lymph node and ileum). Lanes 1–10: Samples of extracted RNA were reverse transcribed then amplified by PCR using specific primers, and the product was examined on an agarose gel. The gel lanes match the corresponding TLR (eg. the lane labelled 1 is TLR1), and the data shown from 1 calf are representative of the 3 calves examined. L: DNA molecular size standards: 400, 300, 200 and 100 bp.

Using immunohistochemistry, primary cultures of bTEC had positive cytoplasmic labeling for pancytokeratin and about 5% had positive cytoplasmic labeling for vimentin (Figure 1). The negative control, prepared by omitting the primary antibody, did not show background staining. Unstimulated bTEC, tracheal tissue, lung tissue, and the positive control (pooled RNA from mesenteric lymph node and ileum) each expressed mRNA for all 10 bovine TLRs examined (Figure 2). Findings in cell cultures, tracheal tissue, and lung tissue were similar for all 3 calves examined. The bTEC and the positive control each showed mRNA expression of IL-17RA (data not shown).

### Effect of agonists on TAP gene expression

**Figure 3:**
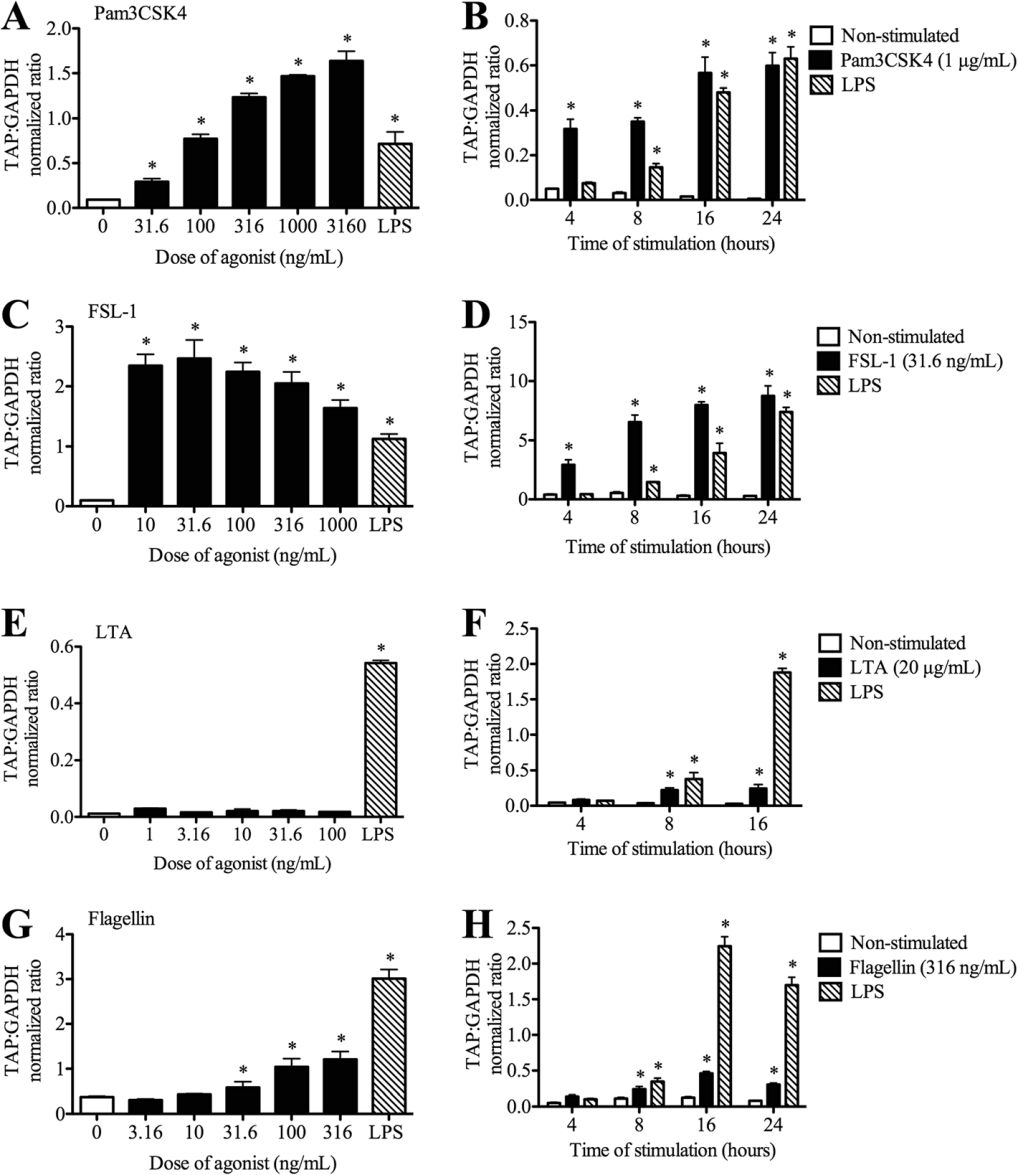
**Effects of Pam3CSK4, FSL-1, lipoteichoic acid and flagellin on tracheal antimicrobial peptide gene expression.** Dose- and time-dependent effects were measured for Pam3CSK4 **(A,B)**, a TLR2/1 agonist; FSL-1 **(C,D)**, a TLR2/6 agonist; lipoteichoic acid (LTA) **(E,F)**, a TLR2/2 agonist); and flagellin **(G,H)**, a TLR5 agonist. For the dose–response studies **(A,C,E,G)**, confluent bTEC were stimulated in triplicate with various concentrations of agonist for 16 h. For the time-course studies **(B,D,F,H)**, confluent bTEC were stimulated in triplicate for 4, 8, 16 or 24 h with the doses of agonist shown. Gene expression of TAP relative to that of GAPDH was measured using real-time RT-qPCR. Lipopolysaccharide (LPS, 0.1 μg/mL) was used in all assays as a positive control and standard. *, significantly different from unstimulated cells (*P* < 0.05).

TAP gene expression was measured in bTEC stimulated with TLR agonists and cytokines. Pam3CSK4, an agonist of the TLR2/1 heterodimer, induced TAP gene expression in a dose-dependent manner from 31.6 ng/mL to a maximal effect at the highest dose tested of 3.16 μg/mL. This effect was significantly different from the non-stimulated control and from LPS-stimulated cells (Figure 3A). At this dose, the magnitude of effect was significantly greater than induction by LPS. The findings were similar in both calves tested, although the magnitude of the response differed. Induction of TAP gene expression was detected as early as 4 h with 1 μg/mL Pam3CSK4 stimulation (Figure 3B).

FSL-1, a TLR2/6 agonist, also showed significant induction of TAP gene expression. A dose of 31.6 ng/mL induced the highest levels of TAP gene expression (Figure 3C). Using this concentration in the time course study, FSL-1 induced significantly greater TAP gene expression compared to the negative control and LPS as early as 4 h of stimulation (Figure 3D). In contrast, lipoteichoic acid, an agonist of the TLR2/2 homodimer, did not significantly induce TAP gene expression in doses ranging from 10–1000 ng/mL (Figure 3E), although the bTEC used in this experiment did respond as expected to LPS (confirming the viability and responsiveness of these cells). In an earlier time-course study using a lipoteichoic acid dose of 20 μg/mL and bTEC sourced from a different calf, significant induction of TAP gene expression was found at 8 and 16 h, albeit at much lower magnitude than for stimulation with LPS (Figure 3F).

In bTEC stimulated with flagellin, a TLR5 agonist, a dose of 316 ng/mL (the highest dose tested) significantly induced TAP gene expression compared to the non-stimulated control. This effect was dose-dependent at both 8 and 16 h of stimulation (Figure 3G). Using this dose, significant induction of TAP gene expression was first detected at 8 h of stimulation (Figure 3F). However, flagellin did not induce significantly higher gene expression than LPS at any of the doses or times tested.

**Figure 4:**
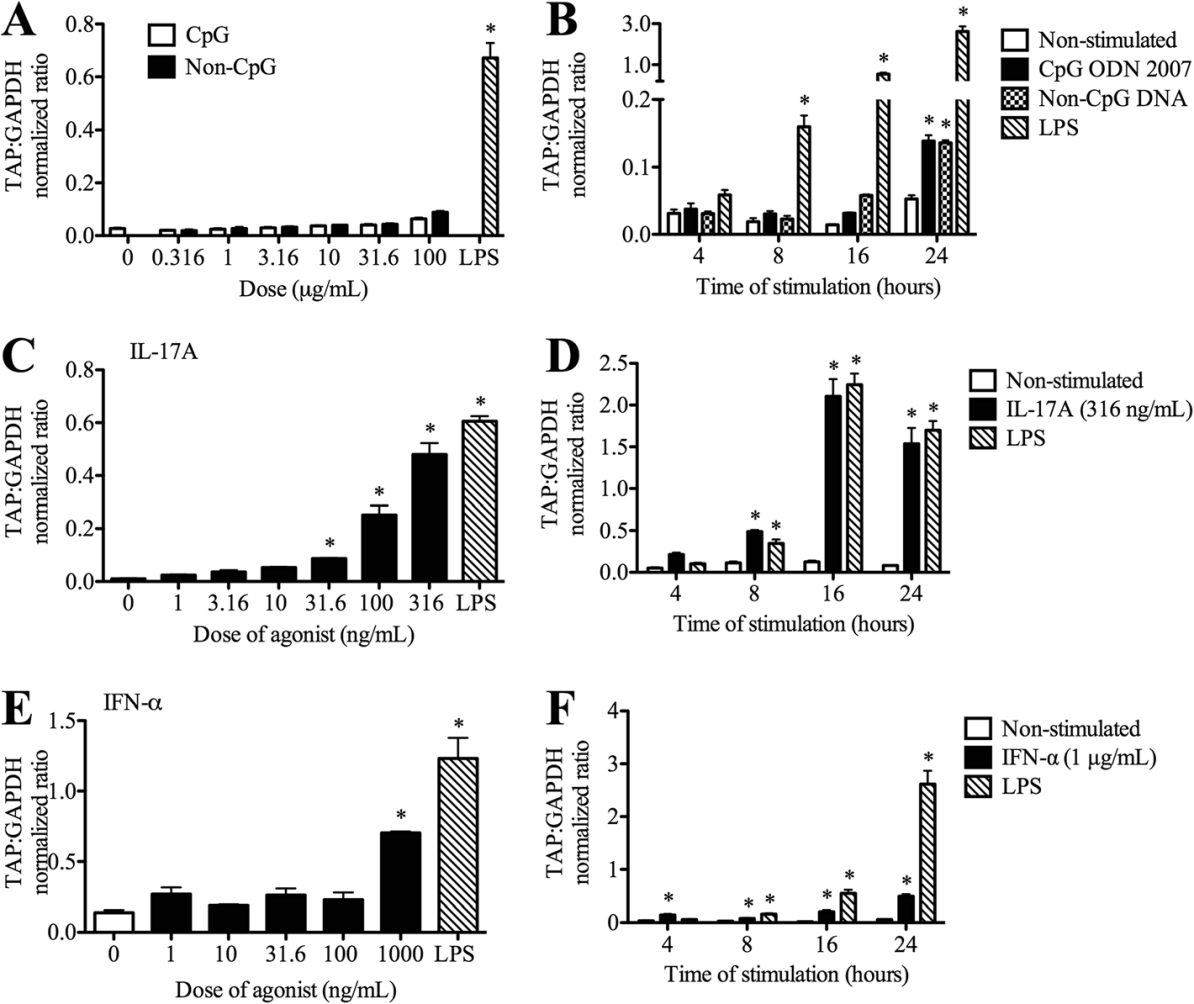
**Effects of CpG oligodinucleotide, interleukin-17A and interferon-α on tracheal antimicrobial peptide gene expression.** Dose- and time-dependent effects were measured for CpG oligodinucleotide **(A,B)**, a TLR9 agonist; interleukin-17A **(C,D)**; and interferon-α **(E,F)**. For the dose–response studies **(A,C,E)**, confluent bTEC were stimulated in triplicate with various concentrations of agonist for 16 h. For the time-course studies **(B,D,F)**, confluent bTEC were stimulated in triplicate for 4, 8, 16 or 24 h with the doses of agonist shown. Gene expression of TAP relative to that of GAPDH was measured using real-time RT-qPCR. Lipopolysaccharide (LPS, 0.1 μg/mL) was used in all assays as a positive control and standard. *, significantly different from unstimulated cells (*P* < 0.05).

Stimulation of bTEC with various doses of CpG ODN 2007 (a bovine/porcine TLR9 agonist) and its non-CpG control did not induce significant TAP gene expression at 8 or 16 h (Figure 4A). A minor non-significant increase was noted for both CpG ODN 2007 and the non-CpG control in the time course study, and a similar non-significant trend was found in the dose–response study. However, CpG did not induce gene expression significantly higher than its non-CPG control at any time (Figure 4B).

Stimulation of bTEC with recombinant bovine IL-17A showed dose-dependent induction of TAP gene expression, to a maximum of 316 ng/mL (the highest dose tested) (Figure 4C). At this dose, IL-17A significantly induced TAP expression after 8 h of stimulation, with a non-significant increase in TAP gene expression as early as 4 h (Figure 4D).

Stimulation of bTEC with recombinant bovine IFN-α showed that a dose of 1000 ng/mL (the highest dose tested) significantly induced TAP gene expression in comparison to the non-stimulated control (Figure 4E). Using this dose of IFN-α, significant induction of TAP gene expression occurred after 4 h of stimulation and remained significantly elevated at later time points. Even though there was a rapid induction caused by IFN-α, it was not higher than LPS (Figure 4F).

**Figure 5:**
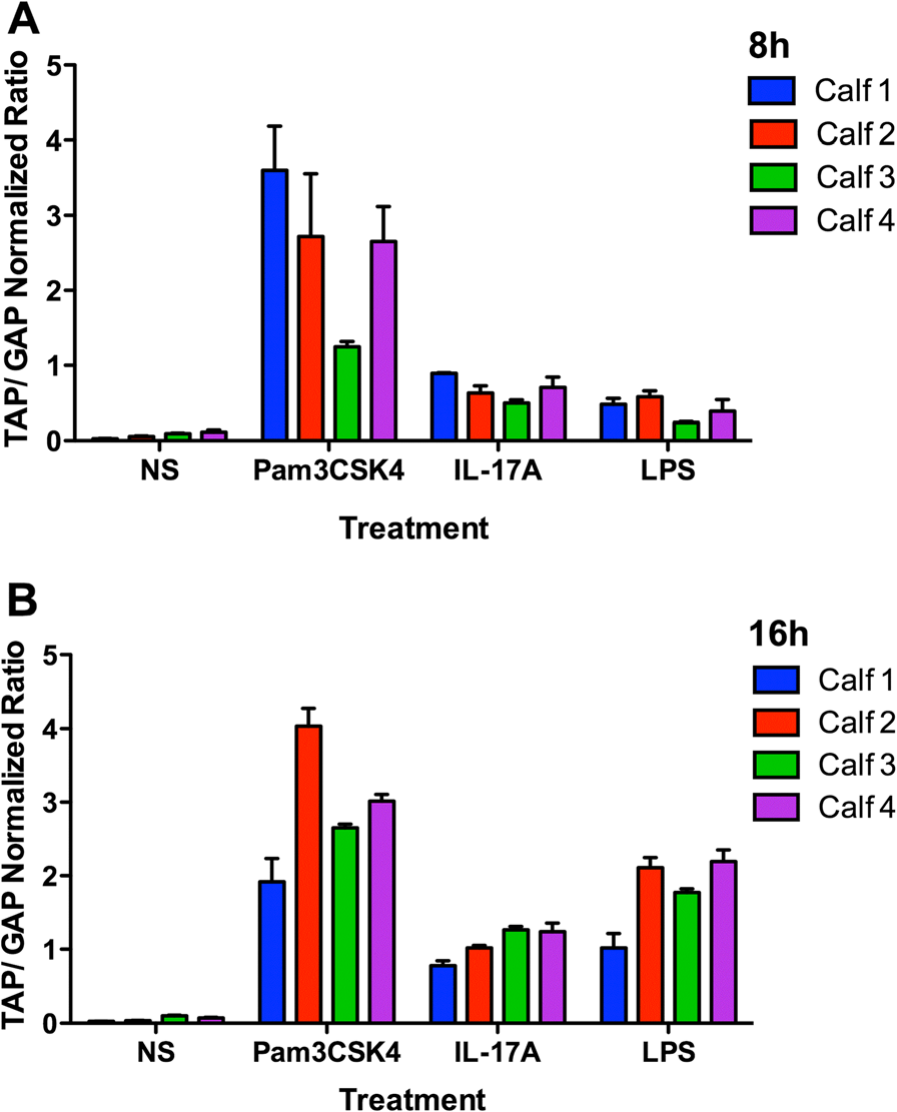
**Comparison of the effects of IL-17A, Pam3CSK4 and LPS on tracheal antimicrobial peptide gene expression.** Confluent cultures of tracheal epithelial cells from 4 different calves were non-stimulated (NS) or stimulated with 1 μg/mL Pam3CSK4, 316 ng/mL IL-17A, or 0.1 μg/mL LPS for 8 h **(A)** and 16 h **(B)** in triplicate. Gene expression was assessed using real-time RT-qPCR. Pam3CSK4 induced significantly higher tracheal antimicrobial peptide gene expression than IL-17A and LPS at both 8 and 16 h (*P* < 0.05). IL-17A induced significantly higher gene expression than LPS at 8 h (*P* < 0.05).

A summative experiment was conducted to compare the effects of Pam3CSK4, IL-17A and LPS on induction of TAP gene expression in bTEC from 4 calves (Figure 5). As above, all agonists had significantly greater effects than no stimulation. Pam3CSK4 induced significantly higher TAP gene expression than did IL-17A or LPS, at both 8 and 16 h of stimulation for all 4 calves examined. IL-17A also induced significantly higher gene expression than LPS after 8 h, but not at 16 h. Differences among the 4 animals tested were significant (*P* < 0.0001).

**Figure 6:**
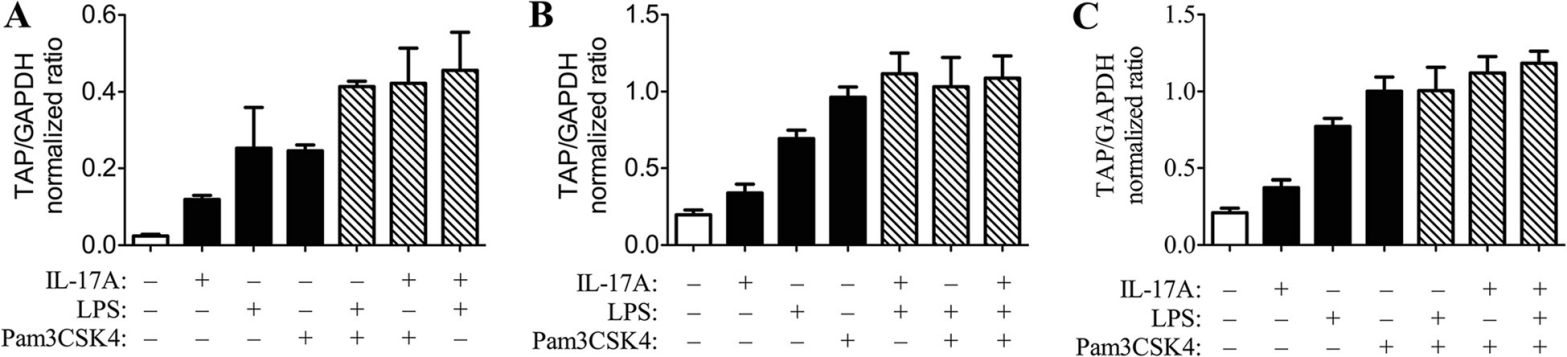
**Effect of stimulation with single agonists compared to combined agonists.** Cultured bovine tracheal epithelial cells were stimulated for 16 h in triplicate with various combinations of 1 μg/mL Pam3CSK4, 316 ng/mL IL-17A, or 0.1 μg/mL LPS. Tracheal antimicrobial peptide gene expression was measured as above. The effects of combined agonists were greater than that of interleukin-17A (IL-17A) alone, but minimally or not different than that of lipopolysaccharide (LPS) or Pam3CSK4 alone. The data shown **(panels A, B and C)** represent 3 studies conducted on different days using cells from different calves.

Three experiments (conducted on different days using cells from different calves) examined the combined effects of LPS, Pam3CSK4 and IL-17A on TAP gene expression (Figure 6). All combinations of agonists tested had a greater effect than IL-17A alone (1-way ANOVA, *P* < 0.05). In one experiment, the combination of LPS and IL-17A had a greater effect than any single agonist (Figure 6A), but the difference was small and not found in the second experiment (Figure 6B). Other combinations of agonists did not have significantly different effects than that of Pam3CSK4 alone or of LPS alone.

## Discussion

The present study investigated factors that induce antimicrobial peptide gene expression in bovine tracheal epithelial cells, with the intent of developing methods to stimulate this response to prevent respiratory disease. The morphology and cytokeratin expression of the primary cell cultures confirmed an epithelial phenotype; occasional cells co-expressed cytokeratin and vimentin, which was consistent with prior reports of tracheobronchial epithelial cells [15]. Initial findings identified gene expression of TLRs 1–10 as well as IL-17RA in bTEC as well as in tracheal and lung tissue, consistent with other findings using primary cultured airway epithelial cells [16]. Histologically, tracheal and lung samples from all calves contained lymphocytes, plasma cells, and neutrophils, albeit in low number. This study examined the presence rather than the level of gene expression, and it is acknowledged that post-translational regulation of these expressed genes would determine functional activity of these cell-surface receptors. However, the subsequent finding that agonists of these receptors induced TAP gene expression implies that functional receptors were expressed. As a result of these findings, agonists for TLRs 1, 2, 5, 6 and 9 and IL-17RA were studied to determine their effect on induction of TAP gene expression.

TLR2 functions either as a homodimer or as heterodimers with TLRs 1 or 6. TLR 2/2 recognizes cell wall components of Gram-positive bacteria such as peptidoglycan and lipoteichoic acid; the TLR 2/2 agonist lipoteichoic acid is a lipid bound to a glycerol phosphate chain. TLR 2/1 recognizes triacylated lipopeptides found in the membrane of Gram-positive and Gram-negative bacteria; Pam3CSK4, a synthetic tripalmitoylated lipopeptide resembling the acylated terminus of bacterial lipoproteins, is an agonist of TLR 2/1. TLR 2/6 recognizes bacterial lipoproteins including FSL-1, an N-terminal fragment of the diacylated LP44 lipoprotein of *Mycoplasma salivarium* [17,18].

Pam3CSK4, the TLR 2/1 agonist, significantly induced TAP gene expression in comparison to non-stimulated controls and to LPS, and this stimulatory effect was observed in a dose- and time-dependent manner. These results are consistent with studies in humans that have shown up-regulation of human β-defensin-2 (hBD-2)—an inducible β-defensin homologous to TAP—in human airway epithelial cells [19]. Stimulation of human tracheobronchial epithelial cells with Pam3CSK4 induced antimicrobial activity against both Gram-positive and Gram-negative bacteria including *Pseudomonas aeruginosa* and *Listeria monocytogenes* [19]. The rapid effect suggests that Pam3CSK4 interacts with TLR 2/1 on the cell surface to trigger intracellular signalling that directly activates transcription of β-defensin genes.

As for Pam3CSK4, stimulation of bTEC with the TLR 2/6 agonist FSL-1 resulted in robust induction of TAP gene expression, in comparison to a non-stimulated control or LPS. In contrast, lipoteichoic acid, the TLR 2/2 agonist, did not induce greater TAP gene expression in the dose–response study than for LPS, and only produced a minimal induction during the time course study. In previous studies, LTA significantly upregulated gene expression of both TAP and lingual antimicrobial peptide in primary bovine mammary epithelial cells. However, when compared to LPS, the effect of LTA in stimulating β-defensin as well as pro-inflammatory cytokine gene expression was significantly lower [5]. Similarly, in a study using human lung epithelial cells, stimulation with LTA showed lower induction of IL-8 gene expression compared to stimulation with LPS [16,20]. Thus, the findings suggest that heterodimerization of TLR2 with TLR1 or TLR6 may augment its function in stimulating this innate immune response.

Flagellin, the major protein of flagella of Gram-negative and Gram-positive bacteria, activates TLR5. In this study, flagellin induced TAP gene expression in bTEC in a dose- and time-dependent fashion. However, it never produced effects as high as or earlier than those induced by LPS. With regards to the in vivo relevance, the major *Pasteurellaceae* bacterial pathogens causing bovine respiratory disease do not possess flagella, suggesting that TLR5 stimulation is not important in pathogenesis of this disease.

Bacterial DNA contains 20 times more unmethylated CG dinucleotides than mammalian DNA, and these CpG motifs are immunostimulatory and recognized by mammalian TLR9 [21,22]. This study used CpG ODN 2007, a bovine/porcine-specific class B ODN, which had no significant effect on TAP gene expression compared to the non-CpG control. This result was consistent with other studies that have shown that CpG ODN had no effect on pro-inflammatory cytokine production in human bronchial epithelial cells [23], although another study found that microbial DNA induced gene expression of hBD-2 in airway epithelial cells by interaction with TLR9 and through activation of NF-κB [24]. CpG may need to be combined with other inflammatory cytokines in order to induce an innate response in epithelial cells; for example, CpG augmented IL-8 production in bronchial epithelial cells co-stimulated with IL-1β and tumour necrosis factor-α, and co-stimulation of mice with class C CpG-ODN2395 and Pam2CSK4 had greater resistance to bacterial challenge than either agonist alone [25-27]. Finally, it is noted that other classes of CpG ODNs were not tested in the present study and may have differing effects.

IL-17A is a potent pro-inflammatory and immunostimulatory cytokine produced by T helper 17 lymphocytes, type 3 innate lymphoid cells and bovine γδ-T cells [28,29]. IL-17 is important for defence of the respiratory tract against bacterial pathogens including *Staphylococcus aureus, Pseudomonas aeruginosa* and *Legionella pneumophila* infections in mice [30-32]. In the present study, IL-17A receptor subunit A gene expression was identified in bTEC, consistent with prior findings in human airway epithelial cells [33]. Exposure of these cells to IL-17A stimulated TAP gene expression in bTEC in a dose- and time-dependent fashion, to a greater degree than did stimulation with LPS. This result is consistent with studies of IL-17A inducing human β-defensin-2 expression in human airway epithelial cells and TAP in bovine mammary epithelial cells [33,34]. These findings suggest that IL-17A induces innate mucosal antimicrobial defences in the bovine respiratory tract.

IFN-α is an antiviral type I interferon. The presence of viral or bacterial nucleic acid within cells stimulates pattern recognition receptors including NOD-like receptors, RIG-I-like receptors, and TLRs 3, 4, 7, 8 and 9, activating interferon regulatory factors to induce IFN-α expression [35]. Secreted IFN-α then interacts with the IFNαβ receptor (IFNAR1/2) to activate the JAK/STAT signalling pathway and induce expression of over 300 immune response genes [36]. IFNAR is expressed in all cell types including tracheal epithelial cells [37]. In the present study, IFN-α induced TAP gene expression in bTEC only at the highest dose tested and not consistently greater than LPS, although there was significant induction of TAP gene expression as early as 4 h.

Significant differences in agonist-induced TAP gene expression were identified when tracheal epithelial cells from different animals were tested in the same experiment. Similarly, replication of experimental findings using tracheal epithelial cells from a different animal yielded qualitatively similar but quantitatively different results. These findings suggest differences among animals in the magnitude of response to the various agonists. Thus, comparison between agonists is only valid for those experiments in which different agonists were compared in the same experiment.

Caution is warranted because in vitro nature of this study would not address the complex interactions between different cell types that occur in the respiratory tract. However, this is advantageous because it allows a specific focus on epithelial cell responses. Further, it is possible that the surface mucus layer present in vivo might alter the effect of agonists, and this in vitro work avoids this complication. Finally, additional studies are needed to determine whether in vivo stimulation of this immunoinflammatory response would protect calves against pneumonia or increase the risk of disease due to a heightened response to bacteria.

The findings of this study are important for understanding host responses to pathogens and for development of methods to stimulate innate immune responses in cattle. Tracheal epithelium is a dynamic sensor of threats entering into the lung and responds to pathogens by providing a physical barrier, a mucociliary escalator, and by secreting various antimicrobial peptides, immununomodulatory proteins, antioxidants and chemokines that attract other cells of both the innate and adaptive immune responses. Tracheal antimicrobial peptide is secreted from the respiratory epithelium in an inducible manner and has bactericidal activity against the major pathogens that cause pneumonia in cattle [1]. Other functions include antiviral effects, CCR6-dependent chemotaxis of immature dendritic cells and T lymphocytes, and epithelial cell proliferation and wound repair [38]. Prior studies have shown that LPS induces TAP gene expression, but the present findings indicate more rapid effects by stimulation of TLR2 and IL-17RA pathways. Although previous studies have shown that using combinations of agonists produced synergistic induction of β-defensins in airway epithelium [34,39,40], the use of combined agonists had minimal or no synergistic effect in this study. Further, combined stimulation may increase the risks of adverse effects, so identifying specific effective pathways is important and therefore was the focus of this work. TAP has antimicrobial activity against the pathogens that cause pneumonia in cattle, but induction of TAP gene expression in bTEC is blocked by stress (modeled by glucocorticoids) or bovine viral diarrhea virus infection. The present study suggests that TLR2 agonists or IL-17A may be of value to stimulate this aspect of innate immunity in immunosuppressed feedlot cattle.

## Abbreviations

bTEC: Bovine tracheal epithelial cells
IFN: Interferon
IL: Interleukin
LPS: Lipopolysaccharide
TAP: Tracheal antimicrobial peptide
TLR: Toll-like receptor
